# The Relevance of Calibration in Machine Learning-Based Hypertension Risk Assessment Combining Photoplethysmography and Electrocardiography

**DOI:** 10.3390/bios12050289

**Published:** 2022-05-01

**Authors:** Jesús Cano, Lorenzo Fácila, Juan M. Gracia-Baena, Roberto Zangróniz, Raúl Alcaraz, José J. Rieta

**Affiliations:** 1 BioMIT.org, Electronic Engineering Department, Universitat Politecnica de Valencia, 46022 Valencia, Spain; jecaser@etsii.upv.es; 2 Cardiology Department, General University Hospital Consortium of Valencia, 46014 Valencia, Spain; lorenzo.facila@uv.es; 3 Cardiovascular Surgery Department, Hospital Clínico Universitario de Valencia, 46010 Valencia, Spain; gracia_juabae@gva.es; 4 Research Group in Electronic, Biomedical and Telecommunication Engineering, University of Castilla-La Mancha, 16071 Cuenca, Spain; roberto.zangroniz@uclm.es (R.Z.); raul.alcaraz@uclm.es (R.A.)

**Keywords:** high blood pressure, hypertension, photoplethysmography, electrocardiography, calibration, classification models, machine learning

## Abstract

The detection of hypertension (HT) is of great importance for the early diagnosis of cardiovascular diseases (CVDs), as subjects with high blood pressure (BP) are asymptomatic until advanced stages of the disease. The present study proposes a classification model to discriminate between normotensive (NTS) and hypertensive (HTS) subjects employing electrocardiographic (ECG) and photoplethysmographic (PPG) recordings as an alternative to traditional cuff-based methods. A total of 913 ECG, PPG and BP recordings from 69 subjects were analyzed. Then, signal preprocessing, fiducial points extraction and feature selection were performed, providing 17 discriminatory features, such as pulse arrival and transit times, that fed machine-learning-based classifiers. The main innovation proposed in this research uncovers the relevance of previous calibration to obtain accurate HT risk assessment. This aspect has been assessed using both close and distant time test measurements with respect to calibration. The k-nearest neighbors-classifier provided the best outcomes with an accuracy for new subjects before calibration of 51.48%. The inclusion of just one calibration measurement into the model improved classification accuracy by 30%, reaching gradually more than 96% with more than six calibration measurements. Accuracy decreased with distance to calibration, but remained outstanding even days after calibration. Thus, the use of PPG and ECG recordings combined with previous subject calibration can significantly improve discrimination between NTS and HTS individuals. This strategy could be implemented in wearable devices for HT risk assessment as well as to prevent CVDs.

## 1. Introduction

High blood pressure or hypertension (HT) is the most significant risk factor for many cardiovascular diseases (CVDs) including cardiac arrhythmias, coronary disease, renal failure and stroke [1]. To this, it must be added that most patients with HT are undiagnosed, as in the early stages and even in the elevated blood pressure stage, HT rarely causes symptoms. For these reasons, regular blood pressure monitoring and the assessment of blood pressure levels is crucial for the prevention and early diagnosis of asymptomatic HT and the study of its evolution over time for diagnosed subjects [2].

Arterial blood pressure (BP) values have two components: systolic blood pressure (SBP), determined by the impulse generated by the contractions of the left ventricle, which indicates how much pressure the blood is exerting against the arterial walls when the heart contracts, and the diastolic blood pressure (DBP), which depends on the resistance of the arteries to the passage of blood and indicates the pressure exerted against the walls when the heart relaxes [3]. BP depends mainly on two variables: the volume propelled by the heart in a unit of time and the resistances offered by the arteries to the passage of blood [4]. In turn, these variables depend on the activity of the autonomic nervous system (ANS), which governs heart rate and the resistance of the arterioles, and, on the other hand, the balance of water and salt filtered through the kidneys, which modulates blood volume.

Traditionally, BP has been measured through invasive as well as non-invasive strategies. Invasive BP measurement has been usually reserved for patient hospitalization, especially in Intensive Care Units (ICUs), where the availability of precise and time-continuous BP measurements is relevant [5]. For non-invasive BP estimation, conventional cuff-based measurement devices, which use oscillometric and auscultation methods, are known to be able to offer adequate accuracy. However, they are not designed to be wearable and only offer a one-off measure. Therefore, they are not compatible with continuous measurement throughout the day due to mobility limitations caused by the device, they are uncomfortable, and their measurement procedure, with the repeated inflation and deflation of the cuff, is somewhat tedious, cumbersome and requires patient attention [6].

Machine Learning classifiers provide many advantages to clinical medicine in general and to biosignal-based HT risk assessment in particular over non-invasive traditional measures, as they can be embedded in wearable devices such as smartwatches, facilitating uninterrupted monitoring throughout the day. This allows both the detection of asymptomatic hypertensive patients and the monitoring of diagnosed patients in their daily lives outside the clinical setting by screening changes in blood pressure.

As a consequence of the above factors, work in this field is focused on the development of cuff-less systems that can provide the user with information about the BP condition in near real time [7]. New wearable devices, such as wristbands or smartwatches capable of monitoring physiological signals that change according to BP level, as the electrocardiogram (ECG) and photoplethysmogram (PPG) do, may facilitate the development of these BP measurement systems [8,9]. The most promising signal is the PPG, an optical measurement technique that can be used to detect changes in blood volume in the micro vascular bed of tissues as a result of cardiac pumping. This technique is based on illumination of the skin measuring changes in light absorption [10]. It is typically implemented with a light-emitting diode (LED) to illuminate the skin and a photodetector to measure the amount of light transmitted or reflected through the skin. The change in tissue light absorption is governed by the amount of protein and hemoglobin in blood and the hemodynamic and physiological condition caused by the change in the properties of the artery [11].

In recent years, many studies have investigated methods to estimate BP using PPG signals. The first work that studied the correlation between the PPG and BP was conducted by Teng and Zhang [12], where a linear regression model was used to evaluate the relationship between four PPG features an BP. Once this relationship was known and established, later studies focused on the use of propagation theory, which extracted key features from ECG and PPG signals, simultaneously collected, for BP estimation.

Propagation features, as pulse transit time (PTT) and pulse arrival time (PAT), have been extensively used in previous works [13,14]. PTT was defined as the time taken for the pressure wave to travel between two arterial sites. Thus, it could be estimated as the time delay between a PPG wavefront measured by two separate sensors located in two distal sites of the body. For its part, PAT was defined as the delay between the electrical activation of the heart (R peak of ECG) and the PPG wavefront at the foot, maximum slope point and peak of the PPG signal, which represents the arrival of the pulse at the measurement location. Cavalcante et al. [15] applied this methodology for the first time using the start and end pulse points of these signals as well as PTT, PAT and pulse wave velocity (PWV) to determine the cardiovascular condition. Furthermore, Chen et al. [16] used ear and toe sensors to determinate PTT and its strong relationship with BP.

Other methodologies used the changes in PPG morphology to estimate BP. In this way, Kurylyak et al. [17] extracted 21 features from the PPG waveform, and demonstrated that PPG features could significantly decrease BP estimation error. Li et al. [18] and Kachuee et al. [19] also combined PAT and morphological parameters of the PPG, improving the accuracy of estimation of BP in comparison to only PAT-based features. After analyzing the different proposed methods to estimate BP, this work introduces a combination of both approaches, propagation theory features and morphological PPG features for enhanced HT risk assessment.

In the studies providing a BP value from PPG recordings, this value was just an estimation, so these methods need medical supervision. Thus, the present work introduces an alternative way to solve the problem of BP classification models with reliability, so that they can automatically provide in a continuous and non-invasive way the subject’s blood pressure condition and can trigger alarms in case of an asymptomatic hypertensive condition. In this same way, Visvanathan et al. [20] used a support vector machine (SVM) to classify BP and Liang et al. [21] used PAT and PPG features and four distinctive classifiers, these being logistic regression, AdaBoost tree, Bagged tree and K-nearest neighbors, for the classification of subjects as a function of BP estimated values.

However, it has been demonstrated that the relationship between the aforementioned PPG-based propagation parameters and BP depends on many physiological factors, such as arterial walls’ thickness and elasticity, age and gender, posture and risk factors of CVDs. Thus, calibration is needed when BP levels from a new subject are going to be evaluated by an automated classification method [22]. Moreover, calibration before measurement is essential to adapt the algorithms to the variations on PPG waveforms, as they are easily corrupted by fluctuations in blood circulation state, affecting the connection between BP and peripheral pulses [23].

The aim of the present study is to develop a classification system for discriminating between normotensive (NTS) and hypertensive (HTS) subjects and to evaluate the need and relevance of per-subject calibration. For this purpose, PPG and ECG simultaneous recordings have been analyzed and processed and propagation features, such as PTT and PAT, combined with other PPG morphological features have been extracted and used to train advanced classification models. The manuscript is organized as follows. Section 2 presents the database, the Machine Learning (ML) method procedure and preprocessing, the analysis techniques and the methods to evaluate the need for calibration. Section 3 presents the results, which will be analyzed in Section 4. Finally, in Section 5, the main scientific contributions of this study are remarked upon.

## 2. Materials And Methods

### 2.1. Materials

In this study, the recordings used were obtained from the MIMIC database, which contains information from ICU patients admitted to Beth Israel Deaconess Medical Center in Boston, USA [24]. This database was chosen as it contains ECG, PPG and invasive BP signals recorded simultaneously in ICU. BP signals in which the systolic or diastolic waves were indistinguishable, ECG signals where QRS morphology was distorted or PPG signals in which the systolic and diastolic waves were indistinguishable and the morphology was distorted were dismissed due to the presence of artifacts.

The BP values were labelled according to the report of the Joint National Committee on the prevention, detection, evaluation and treatment of high blood pressure [25]: as normotensive (NTS) for SBP lower than 120 mmHg, prehypertensive (PHT) for SBP between 120 and 140 mmHg and hypertensive (HTS) for SBP higher than 140 mmHg.

After labelling MIMIC recordings according to SBP values, it was observed that several subjects had stable stretches with different labels. One reason that explained these alterations in SBP values was that all patients were in an ICU, so they may have received treatment or medication that significantly altered SBP levels. Moreover, there were subjects with distant stretches, at different time points, whose SBP values were on the borderline between two labels, so that they had a different label across time even though the changes in SBP were only a few mmHg.

Because of the aforementioned reasons, those subjects with huge alterations of their SBP values (labels including NTS and HTS across time) were dismissed, as they were not suitable to train a classification model aimed at assessing the risk of HT. As a result, subjects maintaining the same label across the recording time were selected. A total of 913 recordings from 69 subjects, 45 being NTS and 24 being HTS, with acceptable signal quality conditions were selected from the MIMIC database. The signals were all recorded simultaneously with a duration of 120 s, a common sampling frequency of 125 Hz and a resolution of 8–10 bits [26].

### 2.2. Signal Preprocessing

The PPG signals were processed by a fourth-order Chebyshev II bandpass filter with cutoff frequencies between 0.5 and 10 Hz [27] to remove minor noises and artifacts caused by sensors’ bad contacts, patient movements or any other interfering physiological activity, such as the respiratory activity, that did not provoke signal dismissing in the previous selection stage of minimum signal quality. Furthermore, the mean value of the filtered PPG was removed to prevent drifts and to allow a better comparison between different signals.

Since the waveform of the PPG signal itself is rather simple and not very informative, the derivatives of the signal were also used to better assess the changes in the signals caused by BP. They represent the velocity plethysmogram (VPG) and the acceleration plethysmogram (APG) and were obtained by applying the first and the second order derivatives, respectively, to the processed PPG signal [28].

The ABP signals which reflected the change in BP over the cardiac cycle were clear and did not require any processing to be applied. For its part, standard preprocessing was applied to each ECG [29]. Thus, they were high-pass filtered with cutoff frequency of 0.5 Hz to remove the baseline, and then low-pass filtered with a cutoff frequency of 50 Hz to reduce high-frequency muscle noise and power line interference, in this case, 60 Hz [29].

### 2.3. Fiducial Points Identification

**Figure 1:**
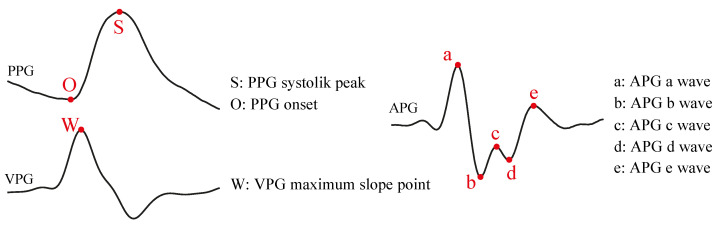
Graphical definition of fiducial points detected from photoplethysmogram (PPG), velocity plethysmogram (VPG) and acceleration plethysmogram (APG) signals.

After signal preprocessing, fiducial points from PPG, VPG and APG were extracted as illustrated in Figure 1. The systolic peaks of the three signals (S, W, a), the onset point of the PPG signal (O), and two local maxima and minimum of the APG signal (b, c, d, e) were extracted [28,30]. Fiducial points in the precessed signals were obtained based on searching local minima and maxima, calculated by establishing threshold and slope criteria in each of the pulses composing every signal.

The maximum systolic blood pressure (SBP) was extracted as the maximum point of each ABP pulse. SBP was used to label every subject as NTS or HTS. Subjects whose selected segments had SBP <130 mmHg were labeled as NTS, and subjects whose selected segments had SBP >130 mmHg as HTS. Finally, for each ECG recording, an R-peak detector based on the phasor transform was applied to the processed ECG signal to obtain the position of each beat [30].

### 2.4. Definition of Discriminatory Features

**Figure 2:**
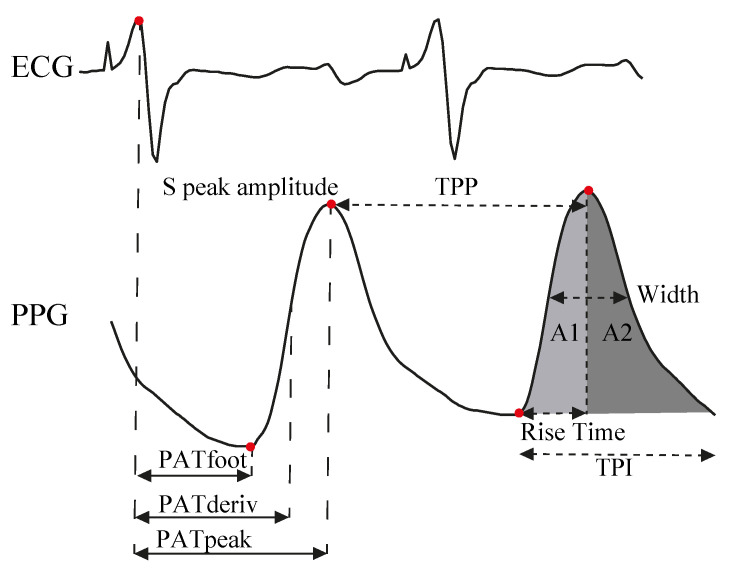
Representation of $PAT_{foot}$, $PAT_{derivate}$ and $PAT_{peak}$ features obtained by the time interval between ECG R-peak and fiducial points of PPG signals as well as PPG morphological parameters: Systolic peak amplitude, TPP, rise time, areas under the pulse, width and TPI.

After the detection of R-peaks in ECG recordings and the fiducial points for each PPG, VPG and APG signals, discriminatory features were defined based on the pulse wave propagation models, such as pulse arrival times (PAT) or pulse transit time (PTT), and other morphological features from the signals that are listed below [28,31,32]. Figure 2 illustrates the definition of the features.

- *PAT*: time interval between R peak and: the O-notch ($PAT_{foot}$), the maximum slope of PPG signal or W peak of VPG signal ($PAT_{derivate}$) and S-peak ($PAT_{peak}$).
- *PTT*: time interval between SBP peak in BP signal to S-peak.
- *Sistolic peak amplitude in PPG*: amplitude from the baseline to S-peaks.
- *Sistolic peak amplitude in VPG*: amplitude from the baseline to W-peaks.
- *TPP*: time interval between two consecutive S-peaks.
- *Time pulse interval (TPI)*: time interval between two consecutive O-notches.
- *Rising time*: time interval between O-notch and systolic peak in PPG signal.
- *Width*: pulse width at half the height of systolic peak height.
- *Pulse area*: integral of the signal between two consecutive O-notches.
- *Area 1*: trapezoidal integration of PPG signal from O-notch to S-peak.
- *Area 2*: trapezoidal integration of PPG signal from S-peak to O-notch.
- *Inflection Point Area (IPA)*: ratio of both areas (A2/A1)
- *a-a*: time interval between two consecutive *a*-peaks in APG signal.
- *Ratios between APG waves with the a-wave*: b/a, c/a, d/a, e/a.
- *Complex APG ratios*: (b−c−d−e)/a, (b−e)/a, (b−c−d)/a, (c+d−b)/a.

### 2.5. Feature Selection

The aim of the feature-selection stage was to select only those features, from the original 23 discriminating parameters, that presented relevant information for solving the classification problem optimally.

Firstly, since all the features were continuous quantitative variables, it was necessary to carry out a normalization, since each one could take on a different range of values and more weight would be given to the variables with higher values, not necessarily being more important. The normalization was carried out using “zscore” centering the variables so that they had zero mean and scaling so that they had unit standard deviation, as represented in the following equation

(1) $$z=\frac{x-\bar{X}}{S},$$

where *x* is a concrete value of a given feature, $\bar{X}$ is the mean of all values of that feature and *S* the standard deviation.

**Figure 3:**
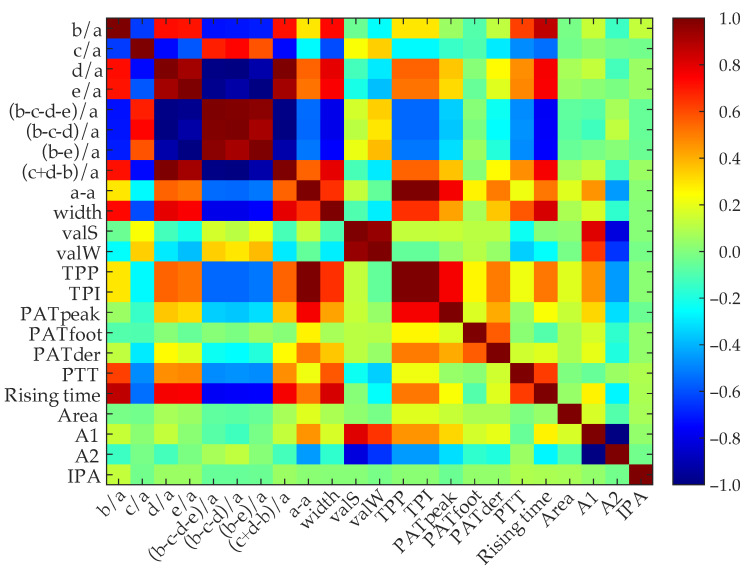
Correlation matrix of the 23 initial discriminatory features used in the study. Dark red values represent higher correlation coefficients and dark blue values represent lower correlation coefficients.

Once the variables were normalized, *ReliefF* algorithm was applied to rank predictors by importance, determining which ones had the best discriminatory power. The key idea of this method is to estimate the quality of predictors according to how well instances near to each other are distinguished, rewarding predictors that give different values to neighbors of a different class [33]. Furthermore, by means of positive and negative correlation, the independence between pairs of variables was analyzed in order to discard those that did not provide new information for the classification task. Figure 3 illustrates the matrix whose entries are the correlation coefficients obtained by matching pairs of variables, so that highly correlated features can be discarded.

After analyzing the correlation matrix and *ReliefF* results, it was decided to remove three complex APG ratios (b−c−d−e)/a, (b−e)/a and (c+d−b)/a as they had high correlation coefficients with other features, as well as the last three *ReliefF* ranked features (TPP, TPI and pulse area), as the deletion of more features worsened classification performance. Finally, after the feature selection, a matrix of 17 normalized features was obtained, which will be used as inputs to train the classification models with ML techniques.

### 2.6. Implementation Details

The experiment was executed under MATLAB (MathWorks, Natick, MA, USA), a scientific and engineering computing software, running on a computer equipped with an Intel i7-8700 CPU @ 3.2 GHz, 16 GB of memory. The implementation for HT risk assessment combining PPG and ECG signals has been based on testing ML classification strategies such as logistic regression, Naive Bayes, discriminant analysis, support vector machines (SVM), k-nearest neighbors (KNN), ensemble classifiers and various types of decision trees [34]. Finally, SVM, Bagging Ensemble classifier and KNN were selected as they provided the highest percentages of classificatory accuracy.

SVM aims at finding the optimal separating hyper-plane between classes by focusing on the training cases that lie at the edge of the class distributions, the support vectors, so only training samples that lie on class boundaries are needed for discrimination [35]. The Bagging technique builds multiple classifiers based on a number of bootstrap samples. The outputs are decided by majority voting [36]. Finally, the KNN classifier obtains the k-nearest neighbors of the data to be classified and, as the Bagging technique, majority voting among the neighborhood is used to decide the output classification [37].

As stated before, the main objective of this study was testing whether HT risk assessment of new subjects could be improved with previous calibration. However, before addressing this goal, the classification of subjects as NTS or HTS, based on discriminant features extracted from PPG and ECG signals, was tested. In so doing, comparison with previous studies without subject-based calibration could be made. The experiment employed a leave-one-out cross-validation strategy. The classification algorithm was applied as many times as segments in the database, using each segment of 2 min in length as a single validation set and all other segments from the same subject, together with the other subjects, as a training set.

Classification performance was assessed with statistical tests for accuracy (*Acc*), sensitivity (*Se*), specificity (*Sp*) and *F1-Score*. *Acc* represented the percentage of correctly assessed PPG segments. *Se* was defined as the ability to detect as positive HTS subjects, whereas *Sp* was defined as the ability to detect as NTS healthy subjects. Finally, *F1-Score* was considered to be the harmonic mean of *Se* and *Acc*. These statistical tests were mathematically computed as

(2) $$Acc=\frac{TP+TN}{TP+TN+FP+FN}$$

(3) $$Se=\frac{TP}{TP+FN}$$

(4) $$Sp=\frac{TN}{TN+FP}$$

(5) $$F1-\mathrm{Score}=\frac{2\cdot Se\cdot Acc}{Se+Acc}=\frac{2\cdot TP}{2\cdot TP+FP+FN}$$

where TN was the number of correctly classified NTS segments, TP the number of correctly classified HTS segments, FN the number of segments that the model predicted as NTS and were actually HTS and FP the number of patients that the model predicted as HTS and were actually NTS.

### 2.7. Need for Calibration of New Subjects

Calibration was defined here as the inclusion of at least one previous measurement of the subject under study in the training set. Aimed at studying the importance of calibration in the classification of new subjects as NTS or HTS, three approaches were taken:

1. Classification performance of new subjects without prior subject-based calibration was studied employing those models providing the best classification results with leave-one-out cross-validation strategy. For this purpose, the analyzed segments of the new subject were only used for validation and the remaining segments from subjects other than the one under study were used to train the model.
2. As a second approach, the effectiveness of calibration to improve classification of new measurements performed later and close in time was studied following the sequel procedure:
  a. Signal segments with a duration of 2 min were divided into 12 sub-segments of 10 s in length.
  b. The sub-segments of all subjects except the one to be analyzed and the first sub-segment of the analyzed subject, acting as the calibration measurement, were used as training dataset and the next sub-segment of the same subject was used for validation.
  c. After its classification, this second sub-segment was introduced in the training dataset, using the next sub-segment for validation. This step was repeated until the 12 consecutive sub-segments of the subject were processed.
  d. This procedure was repeated for all 2 min segments of all subjects in the database.
  This way, a sequential calibration and validation was performed with the idea being to analyze the improvement in classification as the model was gradually calibrated by introducing previous measurements of the same subject very close in time.
3. Finally, the effectiveness of calibration for the classification of distant measurements was studied. To control the distance between measurements, groups of segments of the same patient that were less than 1 h, between 1 h and 6 h, between 6 h and 24 h and more than one day apart were selected. A sequential validation similar to the described for consecutive sub-segments was also followed in this approach in order to study whether classification results improved as the model was calibrated by introducing previous measurements of the same patient far away in time.

The aforesaid three approaches were developed employing the ML classification model that provided the best classification result in a leave-one-out cross-validation strategy.

## 3. Results

Statistical results of classification from the cross-validation strategy to discriminate between NTS and HTS segments are shown in Table 1. As can be seen, all three models provided outstanding classification results, with KNN being the model that obtained the best classification performance with a total accuracy of 93.54%, sensitivity of 92.31%, specificity of 94.35% and F1 score of 91.93%.

Regarding results about the need to calibrate each model to provide the best classification outcome with new subjects, the KNN model was chosen as provided the best classification results with leave-one-out cross-validation. First of all, following the first approach detailed back in Section 2.7, the segments were classified without any previous calibration, in other words, with the training dataset only consisting of segments from other subjects, with each analyzed segment from the subject under study being tested for validation. In this case, classification accuracy with no previous calibration was 51.48%. This proved that hypertension risk assessment of subjects without a prior calibration provided low accuracy results, as has been also reported by previous studies [22,23].

**Figure 4:**
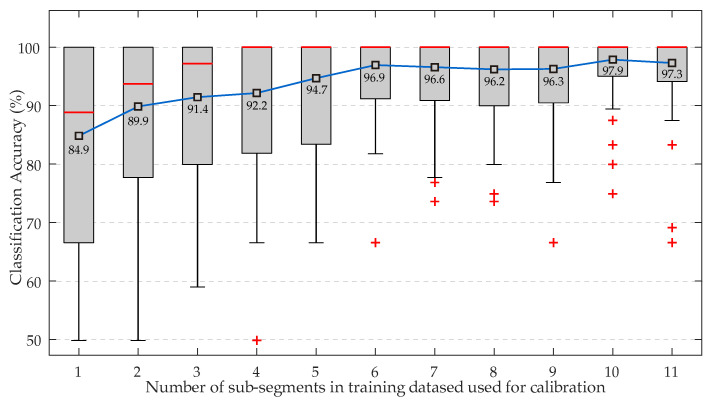
Classification performance provided by the KNN classifier in the discrimination between NTS and HTS individuals. Results obtained for sequential validation of consecutive sub-segments for each of the selected subject segments. In each box, the red line indicates the median, and the bottom and top edges indicate the 15th and 85th percentiles, respectively. The whiskers cover the most extreme data points not considered outliers, and the red symbol (+) stands for outliers. Black squares inside each box indicate mean accuracies.

Next, applying the second approach of Section 2.7, aimed at demonstrating whether poor classification results could be improved with calibration, Figure 4 shows the classification accuracy between NTS and HTS individuals, in the form of box-and-whisker plots, employing sequential validation of consecutive sub-segments. Moreover, the Figure indicates in each square the mean accuracy for all selected subjects according to the number of consecutive sub-segments in the training dataset acting as subject calibration. It can be seen that, with the sole incorporation of one prior close in time sub-segment in the training dataset for calibration, the classification performance increased by 30% with respect to the case without calibration. Furthermore, accuracy improved progressively until it was stabilized above 96%, when more than six prior and close in time sub-segments from the same subject were present in the training dataset.

**Figure 5:**
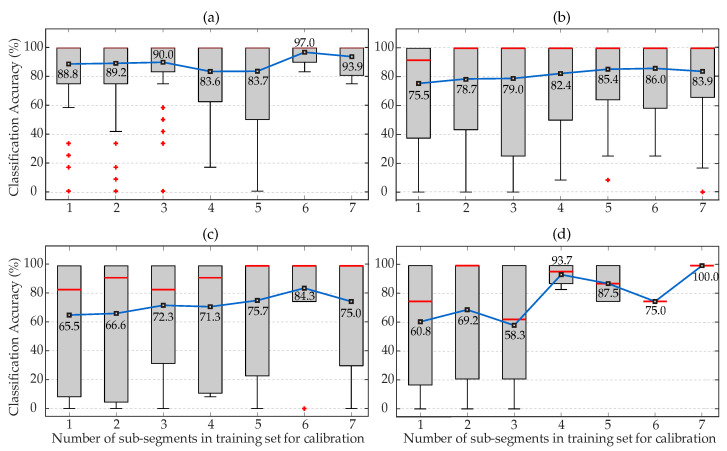
Classification accuracy to distinguish between NTS and HTS individuals using KNN sequential validation of segments with calibration distant from test measurements. (**a**) Distance below 1 h. (**b**) Distance between 1 and 6 h. (**c**) Distance between 6 and 24 h. (**d**) Distance above 24 h. In each box, the red line indicates the median, and the bottom and top edges indicate the 15th and 85th percentiles, respectively. The whiskers extend to the most extreme data points not considered outliers, and the red symbol (+) stands for outliers. Black squares inside each box indicate mean accuracies.

Finally, for the third option defined in Section 2.7, performing calibration distant from measurements, Figure 5 shows classification outcomes of sequential validation with different distances between segments. It was demonstrated that calibration improved the classification task discriminating between NTS and HTS subjects because, as the number of measurements of the same subject in the model increased, so did the accuracy rate. Figure 5 also shows that with calibration and measurement separated by less than 1 h, the model was able to classify with an accuracy beyond 94% from the sixth calibration measurement onwards. As expected, these outcomes decreased as the distance between calibrations and test measurement increased, thus requiring up to five calibration measurements with distances between 6 h and 24 h to obtain classification accuracies above 75%. In any case, the need to perform several calibration measurements to achieve very good classification accuracy with test measurements, which could be many hours or even days away from calibration, does not seem to be a serious limitation. On the other hand, it is worth mentioning that only five subjects had recording lengths longer than five days, so that less stable results in Figure 5d can be considered as normal, because any misclassification would significantly affect the final accuracy. Although the number of subjects was not quite elevated in this last case, the obtained results demonstrate that classification performance was very good even with distances between calibration and test measurement of several days, which is very promising for real-world applications based on embedding these methodologies into wearable devices.

## 4. Discussion

The continuous measurement of BP is of great importance as it facilitates the early detection and prevention of hypertension, being the main risk factor for many CVDs. With the eruption in recent years of the Internet of Things [38] and cuff-less devices that are able to continuously measure and process physiological signals applying artificial intelligence techniques, such as ML and Deep Learning (DL), alternatives to traditional cuff-based single-time BP measurement methods have been proposed. The main signal used in related studies has been the PPG, as its morphological variations are related to the heart’s activity and vascular walls condition, being similar to BP morphology both in frequency and time domains [39]. Furthermore, PPG signal can be acquired by non-invasive low-cost devices as smart watches, obtaining a continuous and real time measurement.

The monitoring of BP through PPG has mainly been studied by two different approaches: (i) addressing the problem of monitoring BP as a regression task estimating systolic and diastolic values; and (ii) addressing the problem of detecting hypertensive subjects as a classification task. In this study, the second approach has been developed, as estimations from the first approach still have serious limitations, so that it is more clinically beneficial to alert hypertensive subjects, acting as a support for clinical decision making.

Tjahjadi et al. [23] proposed the use of KNN technique and PPG signal without ECG, requiring the extraction of 2100 PPG feature points from 2.1 s of data. Their classification results achieved an F1-score of 100% for NTS and PHT patients and 90.80% for HTS patients. Although the authors affirm that this method achieved higher classification performance than other ML and DL methods, obtaining 2100 PPG feature points in such a short period of time required a sampling frequency of 1 kHz, which is a serious drawback for embedding this method in wearable devices, as it significantly increases the amount of data sampled, saved and transmitted which, unavoidably, will involve a considerably high power consumption.

Most studies for HT risk classification use both PPG and ECG signals, as PAT value is directly related to BP value. Although previous works have studied the efficiency of employing PAT as the only parameter to estimate BP [14,40], Liang et al. [21] reported a higher correlation with BP levels by combining PAT with additional PPG features. Dividing the dataset into 70% for training and 30% for validation, the KNN classification model obtained the best performance compared to bagged tree, logistic regression and AdaBoost tree. The F1 scores comparing NTS vs. PHT, NTS vs. HTS and NTS + PHT vs. HTS were 84.34%, 94.84% and 88.49%, respectively. As a consequence, the HT risk-discrimination performance between NTS and HTS was similar to the one achieved in the present study, where F1 score with the KNN classification model was 91.93%, employing leave-one-out cross-validation strategy. Therefore, both in previous studies and in the present work, the KNN classifier has been the best model to assess HT risk, combining PPG recordings and Machine Learning techniques. However, until now, there is no agreement about the discriminant features to be used, since it depends considerably on patient selection and database, mode of acquisition and signal quality.

In recent years, DL approaches have obtained outstanding performance extracting information from images [41,42]. Liang et al. [43] used the continuous wavelet transform of PPG signals and convolutional neural networks to classify BP. The dataset was divided in 80% for training and 20% for testing. The F1 scores for the binary classification comparing NTS vs. PHT were 80.52%, NTS vs. HTS were 92.55% and (NT + PHT) vs. HT trials were 82.95%. The main disadvantages of DL approaches are the requirement of a high computational cost, the extra duration of the training stage and the need for a large number of recordings.

One important consideration introduced by this work, that was not specified in related studies, has been the study of subjects with stable labels of BP. Usually, BP levels vary slightly throughout the day depending on the activities carried out by each person and many other factors, however, each subject would have to be labelled with a single and stable label. For example, any subject cannot be diagnosed as HTS at certain moments of the day, PHT at others and NTS at others. This is a problem when using databases such as the MIMIC, as it consists of recordings from ICU patients that, as a consequence of their unstable condition or the administration of drugs, may have altered and variable SBP levels.

Furthermore, any previous study about hypertension risk assessment has taken into account the relevance of calibration as a factor improving significantly classification results. In this respect, calibration has been only considered in other studies addressing BP estimation in combination with other patient data such as age [44], distance and area of arteries between measure sites or other factors that increase BP as exercise or postural changes [40]. Recently, Schlesinger et al. [45] used convolutional neural networks and PPG signals for BP estimation, achieving a reduction in mean absolute difference of 2.54 mmHg after calibration, using a single 30 s window of PPG signal and the associated BP reading.

The present study has proposed two calibration approaches, trying to improve the poor initial classification accuracy of 51.48% when a new subject entered the method without any previous calibration. The first approach investigated if the method improved classification accuracy when consecutive sub-segments of each subject were used both for calibration and classification, employing sequential validation. The assumption here was the supposed high similarity between calibration measurements and test measurement. Figure 4 showed that the presence of just one calibration sample was enough to increase classification performance more than 30%, which was enhanced even more as the number of calibration measurements raised.

The second approach studied the benefit of calibration for distances between calibration time and measurement time varying from less than 1 h to more than 24 h. This way, it was considered if PPG signal properties from the same patient were kept across time or changed along the day or week. For distances to calibration below 1 h, classification accuracy improved by 30% with just one calibration, keeping these results until more than 6 segments from the same patient were in the training dataset. The improvement of hypertension risk classification decreased slightly as the distance between calibration and measurement increased, although the use of calibration always improved classification results compared to classifying a new uncalibrated subject. Thus, after the fifth calibration, all the experiments provided high accuracy.

These approaches have demonstrated that the properties of each patient’s PPG features were variable over time, as worse results were obtained with measurements distant from calibration than with those very close in time to calibration. Therefore, in order to ensure high classification accuracy, several recalibrations performed at distant recording times and, if possible, in different situations are recommended to accurately asses the risk of HT with PPG and ECG recordings, which can be obtained in a simple way through wearable devices.

Finally, this study has certain limitations that are worth considering. Even though more than 900 recordings were analyzed, the total number of patients was not too large and there was no information available on factors that may imply a higher risk of HT such as age, sex or physical condition. In this respect, Mukkamala et al. [46] studied the age factor in calibration predicting a maximum calibration interval of 1 year for subjects of 30 years of age, that declined linearly to 6 months for subjects at the age of 70, using the PTT as discriminatory feature. In addition, this study has only applied the artificial intelligence technique of ML. Future works will address the application of DL classifiers in order to discern whether they are able to improve hypertension risk assessment of current ML classifiers employing calibrated PPG recordings.

## 5. Conclusions

The combined extraction of discriminant features from PPG and ECG recordings, together with the use of machine learning classification models such as KNN, has been able to perform outstanding hypertension risk assessment in the discrimination between NTS or HTS subjects. The application of per-subject calibration, both in close and distant measurements, has proved its relevance for accurate classification. The implementation of these artificial intelligence techniques in wearable devices would improve the early diagnosis and prevention of cardiovascular diseases associated to hypertension.

## Figures and Tables

**Table 1:** Classification performance to distinguish between NTS and HTS individuals for the best models analyzed with the selected features.

Model	Accuracy	Sensitivity	Specificity	F1-Score
KNN	93.54%	92.31%	94.35%	91.93%
SVM	91.35%	90.93%	91.62%	89.34%
Ensemble	90.69%	82.97%	95.81%	87.66%

## Data Availability

The data supporting reported results and presented in this study are available on request from the corresponding author.
